# Synthesis, Conformational Analysis and ctDNA Binding Studies of Flavonoid Analogues Possessing the 3,5-di-*tert*-butyl-4-hydroxyphenyl Moiety

**DOI:** 10.3390/antiox11112273

**Published:** 2022-11-17

**Authors:** Andromachi Tzani, Eftichia Kritsi, Lamprini Tsamantioti, Ioanna Kostopoulou, Maria-Anna Karadendrou, Panagiotis Zoumpoulakis, Anastasia Detsi

**Affiliations:** 1 Laboratory of Organic Chemistry, Department of Chemical Sciences, School of Chemical Engineering, National Technical University of Athens, Heroon Polytechniou 9, Zografou Campus, 15780 Athens, Greece; 2 Institute of Chemical Biology, National Hellenic Research Foundation, 48, Vas. Constantinou Avenue, 11635 Athens, Greece; 3 Department of Food Science and Technology, University of West Attica, Ag. Spyridonos, 12243 Egaleo, Greece

**Keywords:** flavanones, arylidene flavanone, chalcone, aldol reaction, antioxidant activity, conformational analysis

## Abstract

Flavanones and their biochemical precursors, chalcones, are naturally occurring compounds and consist of privileged scaffolds used in drug discovery due to their wide range of biological activities. In this work, two novel flavanones (**3** and **4**), the arylidene flavanone **5,** and the chalcone **6,** displaying structural analogies with butylated hydroxytoluene (BHT), were synthesized via an aldol reaction. According to the antioxidant activity studies of the synthesized flavanones, the arylidene flavanone **5** was the most potent antioxidant (70.8% interaction with DPPH radical and 77.4% inhibition of lipid peroxidation). In addition, the ability of the synthesized compounds to bind with ctDNA was measured via UV-spectroscopy, revealing that chalcone **6** has the strongest interaction with DNA (K_b_ = 5.0 × 10^−3^ M^−1^), while molecular docking was exploited to simulate the compound-DNA complexes. In an effort to explore the conformational features of the novel synthetic flavanones (**3** and **4**), arylidene flavanone **5,** and chalcone **6**, theoretical calculations were applied and the calculation of their physicochemical properties was also performed.

## 1. Introduction

**Figure 1:**
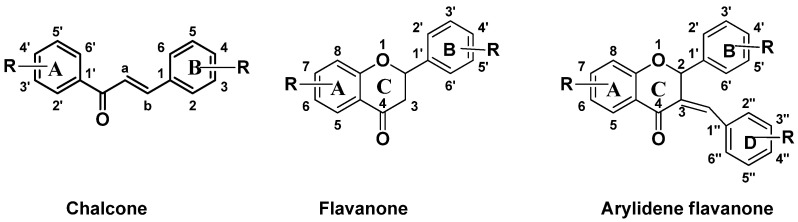
General structure and numbering scheme of the flavanones, chalcones, and arylidene flavanones framework.

Flavanones (2-phenyl-2,3-dihydrochromen-4-ones) and their biochemical precursors, chalcones (Figure 1), belong to the flavonoid family of natural products. Chalcones (1,3-diaryl-2-propen-1-ones) are characterized by the presence of an α,β-unsaturated carbonyl system and an extensive structural diversity. A vast number of chalcones have been isolated from plant species and have been shown to possess antioxidant, anti-inflammatory, antibacterial, and enzyme inhibitory activity, as well as neuroprotective properties [1,2,3,4,5,6].

**Figure 2:**
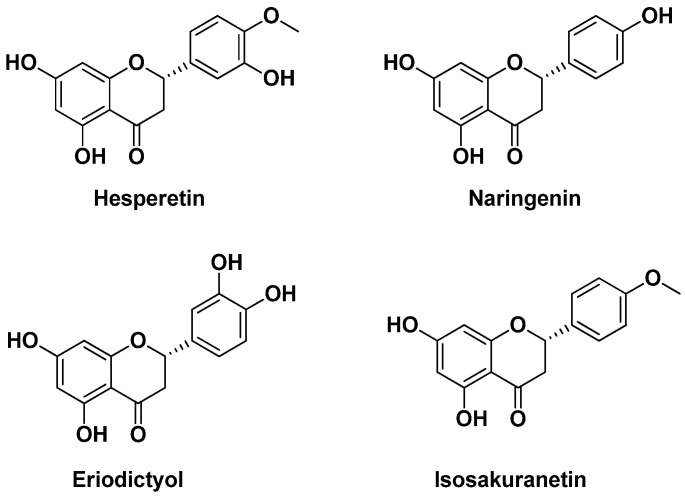
General structure of hesperetin, naringenin, eriodictyol, and isosakuranetin.

Two structural features, the absence of the C2-C3 double bond and the presence of a chiral center at the 2-position, characterize flavanones. Although at first these natural products were considered as “minor flavonoids”, the constantly growing number of flavanones isolated from plants has “promoted” them to major flavonoids. Citrus fruits, tomatoes, and mint are rich sources of flavanones, such as hesperetin, naringenin, eriodictyol, isosakuranetin, and their respective glycosides (Figure 2) [7]. Flavanones are attracting increased attention due to the wide array of biological activity that they possess, such as antioxidant, anticancer, anti-inflammatory, antibacterial, anti-HIV, and others [7,8,9,10]. Furthermore, in the recent work published by the research group of Fang et al., naringenin and the simplest flavanone molecule, with no substituents on the aromatic rings, were shown to be good candidates for the development of antipsoriatic agents, as they showed enhanced skin permeation and anti-inflammatory activity [10,11,12].

Regarding the antioxidant activity of flavanones, it seems that there is an extensive study in the literature investigating their potential activity through several in vitro techniques, such as the DPPH (2,2-Diphenyl-1-picrylhydrazyl), AAPH (2,2’-Azobis(2-amidinopropane) dihydrochloride), ABTS (2,2’-azino-bis(3-ethylbenzothiazoline-6-sulfonic acid), cupric reducing antioxidant capacity, hydroxyl radical, hydroxyl peroxide, or superoxide anion radical assays [13,14,15,16,17,18].The structural features that enhance the activity of flavanones, as well as other phenolic compounds such as flavones, are multiple phenolic groups, especially the ortho-dihydroxy (catechol) configuration at the B ring and the 4-carbonyl group at the C ring [19].

The plausible mechanisms that explain the antioxidant activity of flavonoids have been studied and reviewed by several research groups [7,20,21,22], and it seems that they are greatly affected by the number of the hydroxyl groups and their configuration [23]. One of the best proposed mechanisms of action describes the capacity of flavonoids to scavenge or reduce reactive oxygen species (ROS) by donating a hydrogen atom or an electron [20,24]. Additionally, flavonoids can act as antioxidant agents through an indirect mechanism, in which they interact with proteins or enzymes that play a major role in the defense mechanism of the cells against oxidative stress [20,23].

**Figure 3:**
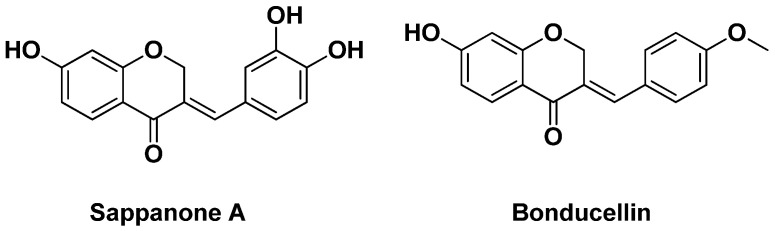
General structure sappanone A and bonducellin.

3-Arylidene flavanones belong to homoisoflavonoids, a small and much less studied group of natural products [25], which possess an exocyclic double bond at position 3 of the heterocyclic ring of the flavanone moiety. Sappanone A (Figure 3), isolated from the heartwood of the plant *Caesalpinia sappan*, is a characteristic example of a 3-arylidene flavanone. It is an inhibitor of viral neuraminidases [26] and also possesses anti-inflammatory [27], antioxidant [28], and anti-ageing [29] activities. Other examples of naturally occurring 3-arylidene flavanones are bonducellin, 8-methoxybonducellin, intricatinol, and eucomin, that were isolated from the methanolic extract of the roots of *Caesalpinia digyna* [30,31].

**Figure 4:**
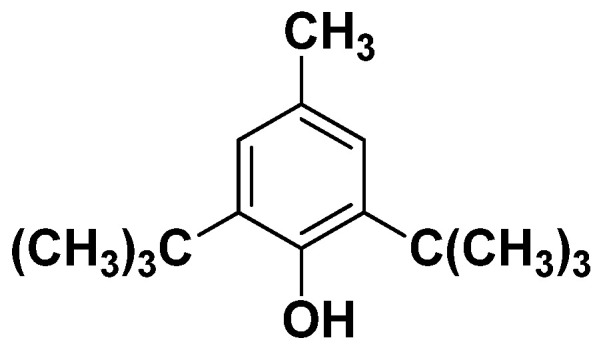
General structure of butylated hydroxytoluene (BHT).

Butylated hydroxytoluene (BHT, Figure 4) is a synthetic phenolic antioxidant widely used as a food additive. BHT inhibits lipid peroxidation and is a powerful free radical scavenger. The characteristic structural features of BHT are the two bulky, electron-donating *tert*-butyl groups at ortho-positions of the phenolic OH. These *tert*-butyl groups increase the lipophilic character of the molecule and stabilize the phenoxy radical formed after the interaction of BHT with an oxidative species and prevent the prooxidant effect of BHT [32].

The synthesis of 2′-hydroxy-chalcone analogues and their hybrids has been the focus of our research [3,4,33], as these compounds are privileged structures and, as such, they are a “canvas” for the synthetic chemist, providing various opportunities for structural modifications. In this context, we set out to investigate the aldol reaction between 2-hydroxy-acetophenone and 3,5-di-*tert*-butyl-4-hydroxybenzaldehyde in order to synthesize chalcones and flavanones displaying structural analogies with BHT.

The antioxidant activity of the synthesized compounds was evaluated in terms of their ability to scavenge the stable free radical DPPH and their ability to inhibit lipid peroxidation. The first technique describes a fast, simple, and cost-effective antioxidant assay based on electron-transfer that produces a violet solution. The free DPPH radical, which is stable at room temperature, is reduced in the presence of an antioxidant compound, decolorizing the solution. The interaction of the tested molecules with the free radical indicates their scavenging ability in an iron-free system [4,34]. The second method is based on the ability of the compounds to inhibit lipid peroxidation of linoleic acid, induced by AAPH, which is a thermal free radical producer. This technique has been developed as a quick and reliable assay. AAPH generates free radicals in the solution, which cause the oxidation of linoleic acid, and the technique estimates how effectively antioxidants protect against lipid peroxidation in vitro [4,34].

Furthermore, the ability of the compounds to bind with ct-DNA was evaluated. Conformational analysis and the prediction of the main physicochemical characteristics of the compounds were also performed.

In the pharmaceutical industry, DNA is one of the most studied pharmacological targets, owing to its major role in cell functions. Small molecules are able to bind with DNA and alter its function depending on the way that each molecule interacts with the double helical structure of DNA [35,36]. There are two different ways of DNA-drug binding: the covalent and the non-covalent. The covalent mode of binding has an irreversible effect on DNA function, since it leads to the total inhibition of DNA processes and, therefore, cell death [35]. The non-covalent mode of binding is reversible, is considered less cytotoxic than the covalent mode, and it includes three different types of interaction: intercalation, groove binding, and external static electronic effects [35,36,37,38]. Compounds that act as intercalators stack between adjacent DNA base pairs. Intercalation occurs perpendicular to the axis of the helix, without disrupting the form of the DNA; however it affects many biological functions. Compounds that act as groove-binding drugs can either bind to the minor or to the major groove by van der Waals interactions and hydrogen bonds, resulting to no or small structural modifications of the DNA core. Compounds that act as external ligands interact electrostatically with the DNA phosphate backbone, which is negatively charged. Since the interactions with DNA have attracted scientific interest, many techniques have been established in order to study drug-DNA binding. In this context, UV-visible absorption spectroscopy was employed to investigate the ability of the synthesized compounds to bind with DNA, while the molecular docking technique was also utilized for a more in depth study of this phenomenon.

## 2. Materials and Methods

### 2.1. Synthesis and Characterization

The synthesized compounds were structurally elucidated using a Varian 300 MHz NMR spectrometer (Palo Alto, CA, USA) using DMSO-d_6_ and CDCl_3_ at 99.9% D. Coupling constants (*J*) are expressed in hertz (Hz). Chemical shifts (*δ*) are reported in parts per million (ppm) units relative to the reference (TMS). Mass spectrometry (MS) analysis was performed on a Varian 500 MS ion trap mass spectrometer using electrospray ionization (ESI). NMR and MS spectra of compounds **3**–**6** are available in the Supplementary materials.

Melting points are determined on a Gallenkamp MFB-595 melting point apparatus (London, UK) and are uncorrected.

All commercially available starting materials and solvents were used without further purification.

#### General Procedure for the Synthesis of Flavanones 3–5

Equimolar amounts of 2-hydroxy-acetophenone (**1a**–**c**) and 3,5-di-(*tert*-butyl)-4-hydroxybenzaldehyde (**2**) are added to dry methanol saturated with hydrogen chloride gas. The mixture is stirred at room temperature. After the completion of the reaction, the product precipitates in the reaction mixture and, after cooling, the solid is filtered and obtained upon crystallization.

6-bromo-2-(3,5-di-*tert*-butyl-4-hydroxyphenyl) chromane-4-one (**3**)

According to the general procedure, equimolar amounts of 5-bromo-2-hydroxy-acetophenone (**1b**) (300.0 mg, 1.39 mmol) and 3,5-di-(*tert*-butyl)-4-hydroxybenzaldehyde (**2**) (326.9 mg, 1.39 mmol) are added to 7.60 mL of dry methanol saturated with hydrogen chloride gas. The mixture is stirred for 4 h at room temperature. The product precipitates in the reaction mixture and, after cooling, the solid is filtered off and washed with methanol. The product is obtained as a light-yellow powder. Yield: 93%; m.p.: 240–243 °C; **^1^H NMR (300 MHz, CDCl_3_)** *δ* ppm 8.05 (d, *J* = 2.5 Hz, 1H, H-5), 7.58 (dd, *J* = 8.7, 2.5 Hz, 1H, H-7), 7.27 (s, 2H, H-2′ and H-6′), 6.97 (d, *J* = 8.8 Hz, 1H, H-8), 5.39 (m, 2H, H-2 and 4′-OH), 3.15 (dd, *J* = 17.0, 13.6 Hz, 1H, H-3a), 2.86 (dd, *J* = 17.0, 2.8 Hz, 1H, H-3b), 1.47 (s, 18H, 2xC(CH_3_)_3_). **^13^C NMR (75 MHz, CDCl_3_)** *δ* ppm 191.3, 160.7, 154.5, 138.6, 136.4, 129.5, 128.6, 123.5, 122.2, 120.3, 114.1, 80.7, 44.1, 34.5, 30.2 **MS**: *m*/*z* = 431 [M + 1]^+^ and 433 [M + 1 + 2]^+^.

6-chloro-2-(3,5,-di-*tert*-butyl-4-hydroxyphenyl) chromane-4-one (**4**)

According to the general procedure, equimolar amounts of 5-chloro-2-hydroxy-acetophenone (**1c**) (270.2 mg, 1.58 mmol) and 3,5-di-(*tert*-butyl)-4-hydroxybenzaldehyde (**2**) (370.3 mg, 1.58 mmol) are added to 8.62 mL of dry methanol saturated with hydrogen chloride gas. The mixture is stirred for 4 h at room temperature under an inert atmosphere. The product precipitates in the reaction mixture and, after cooling, the solid is filtered off and obtained upon recrystallization from methanol–dichloromethane as a pale brown powder. Yield: 70 %; m.p.:231-234; **^1^H NMR (300 MHz, CDCl_3_)** *δ* ppm 7.88 (d, *J* = 2.3 Hz, 1H, H-5), 7.43 (dd, *J* = 8.8, 2.3 Hz, 1H, H-7), 7.27 (s, 2H, H-2′ and H-6′), 7.01 (d, *J* = 8.8 Hz, 1H, H-8) 5.39 (m, 2H, H-2 and 4′-OH), 3.17 (dd, *J* = 16.9, 13.6 Hz, 1H, H-3a), 2.88 (dd, *J* = 16.7, 2.4 Hz, 1H, H-3b), 1.49 (s, 18H, 2xC(CH_3_)_3_). **^13^C NMR (75 MHz, DMSO-d_6_):** *δ* (ppm) 191.6, 154.8, 147.8, 139.6, 136.1, 129.7, 127.1, 125.7, 123.9, 123.1, 120.9, 80.5, 43.5, 30.7, 30.6. **MS**: *m*/*z* 387.6 [M + 1]^+^ and 389.7 [Μ + 1 + 2]^+^.

3-(4′-hydroxy-3′,5′-di-*tert*-butylbenzylidene)-4″-hydroxy-3″,5″-di-*tert*-butyl-flavanone (**5**)

According to the general procedure, equimolar amounts of 2-hydroxy-acetophenone (**1a**) (204.6 mg, 1.50 mmol) and 3,5-di-(*tert*-butyl)-4-hydroxybenzaldehyde (**2**) (352.0 mg, 1.50 mmol) are added to 8.18 mL of dry methanol saturated with hydrogen chloride. The mixture is stirred for 4 h at room temperature under an inert atmosphere. After the reaction is completed, the product precipitates and, after cooling, the solid is filtered off and is recrystallized from methanol–dichloromethane as a light-yellow powder. Yield: 65%; m.p.: 196–199 °C (m.p. [39]: 206–207 °C); **^1^H NMR (300 MHz, CDCl_3_)** *δ* ppm 8.07 (s, 1H, C=CH′), 7.94 (dd, *J* = 7.8, 1.4 Hz, 1H, H-5), 7.38 (td, 1H, H-6), 7.25 (s, 2H, H-2″ and H-6″), 7.09 (s, 2H, H-2′ and H-6′), 6.95 (t, *J* = 7.5 Hz, 1H, H-7), 6.89 (d, *J* = 8.2 Hz, 1H, H-8), 6.55 (s, 1H, H-2), 5.49 (s, 1H, 4″-OH), 5.19 (s, 1H, 4′-OH), 1.37 (s, 18H, 2x[-C(CH_3_)_3_]), 1.31 (s, 18H, 2x[-C(CH_3_)_3_]). **^13^C NMR (75 MHz, DMSO-d_6_)** *δ* (ppm) 181.2, 158.4, 156.2 154.1, 139.8, 139.1, 138.9, 136.1, 129.0, 128.3, 127.9, 126.8, 124.9, 124.0, 121.9, 121.7, 118.6, 78.4, 34.5, 34.5, 30.1, 29.9 **MS**: *m*/*z* = 569.4 [Μ + 1]^+^.

2′,4-Dihydroxy-3,5-di-(*tert*-butyl)-chalcone (**6**)

Equimolar amounts of 2-hydroxy-acetophenone (**1a**) (300.0 mg, 2.2 mmol) and 3,5-di-(*tert*-butyl)-4-hydroxybenzaldehyde (**2**) (516.4 mg, 2.2 mmol) are added to 12.0 mL of dry MeOH saturated with hydrogen chloride. The mixture is stirred for 3 h at 40 °C under an inert atmosphere. After the reaction is completed, the product precipitates and, after cooling, the solid is filtered off and recrystallized from methanol–dichloromethane as a light-yellow powder. Yield: 35%; m.p.: 170–173 °C (m.p. [39]: 173–174 °C); **^1^H NMR (300 MHz, CDCl_3_)** *δ* ppm 12.97 (s, 1H, 2′-OH), 7.92 (d, *J* = 15.3 Hz, 1H, H_a_, overlapping with the signal of H-4′), 7.95–7.90 (m, 1H, H-4′), 7.49 (d, *J* = 14.0 Hz, 1H, H_b_, overlapping with the signals of H-2, H-6, H-6′), 7.51–7.47 (m, 3H, H-2, H-6, H-6′), 7.03 (d, *J* = 8.4 Hz, 1H, H-3′), 6.95 (t, *J* = 7.6 Hz, 1H, H-5′), 5.64 (s, 1H, 4-OH), 1.52 (s, 18H, 2x[-C(CH_3_)_3_]). **^13^C NMR (75 MHz, CDCl_3_)** *δ* ppm 193.7, 163.5, 156.9, 147.1, 136.6, 136.0, 129.5, 126.3, 126.1, 120.2, 118.7, 118.6, 116.7, 34.4, 30.2 **MS:** *m*/*z* = 353.7 [M + 1]^+^, 354.6 [M + 2]^+^.

### 2.2. In Vitro Assays

#### 2.2.1. DPPH Radical Scavenging Ability

The scavenging effect of the synthesized compounds on the DPPH radical was evaluated following the method described in references [4,33]. The results are presented in Table 1.

#### 2.2.2. Inhibition of AAPH Induced Linoleic Acid Oxidation

The ability of the synthesized compounds to inhibit AAPH induced linoleic acid oxidation was evaluated following the method described in the reference [40]. The results are presented in Table 1.

#### 2.2.3. DNA Binding Studies Using UV-Vis Spectroscopy

The ability of the synthesized compounds to bind with DNA was evaluated following the method described in the reference [37], slightly modified. Lyophilized calf-thymus DNA (ctDNA) was dissolved in Tris-HCl buffer solution (10 mM and pH 7.4) to a concentration of 10mM and left overnight at 4 °C. The absorbance ratio A_260_/A_280_ of DNA was found in the range of 1.8–1.9, indicating that the DNA is sufficiently free of protein. The concentration was determined from the absorbance at 260 nm, using an extinction coefficient of 6600 M^−1^ cm^−1^. The tested compounds were dissolved in DMSO to a concentration of 10 mM. An amount of 1 μL of the prepared solutions was added to 999 μL solutions of ctDNA of different concentrations (0–100 μΜ), incubated for 5 min at 37 °C, and the absorbance of the occurring samples was measured. The binding constant K_b_ was determined via the Benesi-Hildebrand Equation (1):

(1) $$\frac{1}{A-A_{o}}=\;\frac{1}{A_{c}-A_{o}}\;+\;\frac{1}{\left(A_{c}-A_{o}\right)\times \;K_{b}\times \;\left[DNA\right]}$$

All tests were undertaken on three replicates, and the results presented in Table 2 were averaged and compared with the standard Methyl Green.

### 2.3. Molecular Docking

The study of the interaction mode and binding affinity docking studies has been performed with the crystal structure of the DNA (PDB ID: 1bna), was obtained from the B-DNA dodecamer sequence (PDB ID: 1BNA), and was obtained from the protein data bank (https://www.rcsb.org, accessed on 19 October 2022). In order to perform blind docking calculations between the tested compounds and the DNA sequence, MGL tools 1.5.4 (The Scripps Research Institute, La Jolla, CA, USA) were employed. Receptor (DNA) and ligand (synthesized compounds) files were provided using AutoDock Tools (The Scripps Research Institute, La Jolla, CA, USA). The docking process was executed after the removal of water molecules and the addition of polar hydrogen atoms and Kollman charges. The receptor and the ligand were enclosed in a 126x126x126 grid box with a grid spacing of 0.375 Å. Docking calculations were performed via the Lamarckian genetic algorithm, while all other parameters were on default settings. For each docking case performed, the lowest energy docked conformation, according to the AutoDock (The Scripps Research Institute, La Jolla, CA, USA) scoring, was selected as the binding mode. The docked structures and interactions were visualized using the Discover Studio visualizer 4.1 software (BIOVIA, Dassault Systèmes, Vélizy-Villacoublay, France) and the Pymol molecular graphics program.

### 2.4. Conformational Analysis

The synthesized compounds were initially sketched in 2D and were subjected to energy minimization (Macromodel program—Schrödinger Release 2020-3: MacroModel; Schrödinger, LLC: New York, NY, USA, 2020) [41] with an OPLS_2005 force field [42] in chloroform (CHCl_3_) solvent. For all synthesized compounds, a local minimum was identified, applying the following parameters: PRCG (Polak-Ribier Conjugate Gradient) method [43], maximum number of iterations equal to 10,000 and 0.001 kcal · mol^−1^ · Å^−1^ as energy tolerance.

A conformational search (Macromodel program - Schrödinger Release 2020-3: MacroModel; Schrödinger, LLC: New York, NY, USA, 2020) [41] was performed to produce random conformers for all synthesized compounds, using the mixed torsional/low mode sampling method. In the present method the torsional sampling (MCMM method), which includes random changes in torsion angles and/or molecular position, is combined with the low steps derived from the LMOD method. This method is effective, and its main advantage is that rings and variable torsion angles are not specified. Regarding the described conformational search, 1000 and 100 steps were set as the maximum number of steps and the number of steps per rotatable bonds, respectively. The energy window was defined as equal to 100 kJ · mol^−1^ and the RMSD cut-off was 0.5 Å.

A coordinate scan (Macromodel program - Schrödinger Release 2020-3: MacroModel; Schrödinger, LLC: New York, NY, USA, 2020) [41] was implemented to record the favorable torsion angles, corresponding to conformers that present the minimum energy and energy limitations of the novel synthesized flavanones (compounds **3** and **4**). During this process, conformations were created by varying specified torsion angles. An increment of 5° (compounds: **3** and **4**) was applied for a single bond rotation, respectively.

Hydrophilic and hydrophobic surfaces were calculated for the low energy conformers, using the Maestro surfaces panel [44].

### 2.5. Calculation of Physicochemical Properties

Molecular descriptors and properties of the examined compounds were generated utilizing the QikProp program—Schrödinger Release 2020-3: QikProp; Schrödinger, LLC: New York, NY, USA, 2020 [45] of Schrödinger Suite (Release 2020-3).

## 3. Results

### 3.1. Chemistry

**Scheme 1:**
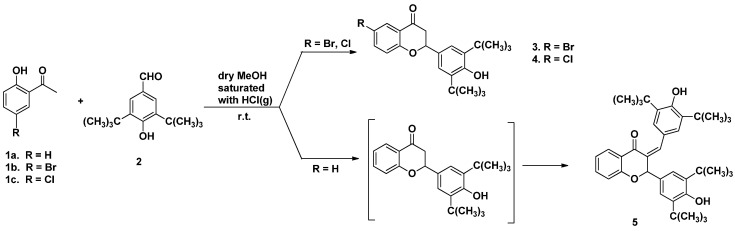
Synthetic procedure of flavanones **3**–**4** and arylidene flavanone **5**.

The aldol reaction, and especially the Claisen-Schmidt type between an acetophenone and a benzaldehyde, is commonly performed in alkaline conditions (for example, aqueous KOH in ethanol). However, in alkaline conditions, 3,5-di-*tert*-butyl-4-hydroxybenzaldehyde (**2**) is in equilibrium with the corresponding quinone methide anion, which predominates and significantly lowers the electrophilic character of the benzaldehyde [39,46]. As is very nicely explained in the work of Adams [40], in acidic conditions, the protonation of the aldehyde group enhances its electrophilic character and the benzenoid tautomers are more stable than the quinoid tautomer, which is unreactive. Thus, we decided to perform the reaction in acidic conditions and used dry methanol saturated with hydrogen chloride (Scheme 1).

The reactions were performed using equimolar amounts of the reactants and stirring at room temperature (approximately 22 °C) for 4 h. In all cases, a single product was obtained, as indicated by NMR spectroscopy, in the form of a solid, which precipitated from the reaction medium. Although we chose to further purify the products via recrystallization, this step could have been omitted, as the purity of the products was satisfactory after the filtration of the solid and washing with methanol.

The presence of a halogen substituent at the starting 2-hydroxy-acetophenone (**1b** and **1c**) was crucial for the progress of the reaction: in these cases, the obtained products were exclusively the flavanones **3** and **4**, in very good yields (>70% after recrystallization). On the other hand, the reaction of 2-hydroxy-acetophenone (**1a**) with 3,5-di-*tert*-butyl-4-hydroxybenzaldehyde (**2**) produced only the corresponding arylidene flavanone **5**, which is the product of a further condensation reaction between the intermediate flavanone and another molecule of 3,5-di-*tert*-butyl-4-hydroxybenzaldehyde.

In all cases, the reaction proceeds via an aldol condensation to form a chalcone, which was not isolated, followed by an intramolecular Michael addition reaction to produce the flavanone. The presence of the electron-withdrawing Br or Cl substituents at the aromatic ring of the 2-hydroxy-acetophenone did not prevent the hydroxyl group from acting as a Michael donor. The same observation has been reported by Zhang et al. [47] who studied the enantioselective cyclization of 2′-hydroxy-chalcone to a flavanone in the presence of organocatalysts.

The arylidene flavanone **5** was the only product when 2-hydroxy-acetophenone (**1a**) was the reactant. No traces of the corresponding chalcone or flavanone were observed at the conditions that we used to perform the reaction (MeOH saturated with HCl (g), stirring at room temperature for 4 h). However, Adams et al. [39] reported all of the three possible reaction products under analogous conditions (EtOH saturated with HCl (g), stirring at room temperature for 2 h). A plausible explanation is the longer reaction time of our experiments, as well as the different isolation procedures (arylidene flavanone **5** was insoluble in the reaction mixture and precipitated, thus the reaction equilibrium shifted towards this product).

**Scheme 2:**
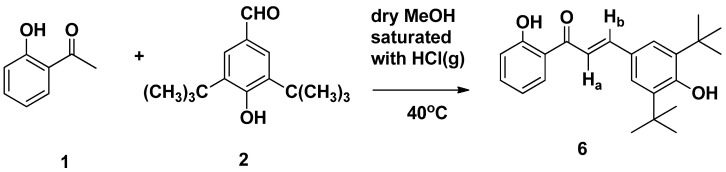
Synthetic procedure of chalcone **6**.

Although the formation of the two new flavanones, **3** and **4**, as well as the arylidene flavanone **5,** in high yields and purity exhibited very gratifying results, we were still interested in synthesizing chalcone **6** (Scheme 2), thus we performed the reaction between **1a** and **2** at 40 °C. After 3 h, a yellow solid precipitated, which was filtered and washed with ethanol. The NMR spectrum verified the structure of the desired chalcone. To our knowledge, only two references exist in the literature concerning the synthesis of this molecule, indicating that its preparation is not trivial [39,48].

The ^1^H NMR spectrum of chalcone **6** has the characteristic peaks attributed to the a,b-unsaturated carbonyl system, which confirms the structure of the synthesized compound. The signals of the vinylic protons H_a_ and H_b_ appear as doublets at 7.92 ppm and 7.49 ppm, respectively, with *J* ~ 14–15 Hz, indicative of the *E*-geometry of the double bond [3]. In addition, the signal of the 2′-OH proton is present at low field, 12.97 ppm, indicating a strong hydrogen bond with the carbonyl oxygen.

### 3.2. Antioxidant Activity

The antioxidant activity of the new flavanones **3** and **4,** the arylidene flavanone **5**, as well as the chalcone **6,** was evaluated by measuring the ability of the compounds to scavenge the stable free radical DPPH (2,2-Diphenyl-1-picrylhydrazyl) and the ability to inhibit the AAPH induced lipid peroxidation of linoleic acid. The results are presented in Table 1. Trolox was used as the reference compound.

The best DPPH radical scavenging ability is shown by arylidene flavanone **5** (70.8% after a 30 min interaction with a DPPH radical), probably due to the presence of two 3,5-di-*tert*-butyl-4-hydroxyaryl-substituents, and it is marginally reduced from 30 to 60 min (60.2%). Flavanones **3** and **4**, which possess one 3,5-di-*tert*-butyl-4-hydroxyaryl-substituent on the aromatic ring B and one halogen substituent at position 6 of the aromatic ring A, exhibit significantly lower activity (29.3% and 32.0% after a 30min interaction with a DPPH radical, respectively) than arylidene flavanone **5**. However, flavanone **4,** possessing a chlorine substituent, is slightly more potent than flavanone **3,** which possesses a bromide one, probably due to the highest electronegativity of chlorine. Furthermore, it seems that the antioxidant activity of flavanone **4** is slightly increased during the time (from 32.0% after 30min interaction to 39.6% after 60 min), while the activity of flavanone **3** does not seem to be time dependent.

As far as chalcone **6** is concerned, it shows a good DPPH radical scavenging activity (61.1% after a 30min interaction with a DPPH radical), higher than flavanones **3** and **4**, which also possess one 3,5-di-*tert*-butyl-4-hydroxyaryl-moiety. 2′-Hydroxy-chalcone analogues that have been synthesized and studied in our previous works [3,4], especially those without other hydroxyl substituents at rings A and B, show very low or zero interaction with the DPPH radical. The 2′-OH is not able to efficiently interact with DPPH, as it participates in a strong hydrogen bond with the neighboring carbonyl group. Therefore, the enhanced activity of chalcone **6** should be attributed to the presence of the 3,5-di-*tert*-butyl-4-hydroxyaryl-substituent.

Regarding the lipid peroxidation inhibitory ability of the tested compounds, again, arylidene flavanone **5** demonstrated the best activity (77.4%), indicating that the presence of a 3,5-di-*tert*-butyl-4-hydroxyaryl moiety enhances anti-lipid peroxidation, since flavanones **3** and **4**, which possess only one 3,5-di-*tert*-butyl-4-hydroxyaryl-substituent on the aromatic ring B, exhibit lower inhibitory activity (21.1 and 59.3% respectively). Interestingly, flavanone **4**, bearing a chloro-substituent, presents a significantly higher ability to inhibit lipid peroxidation than flavanone **3**, which is in accordance with the DPPH results, concluding that the insertion of the chloro substituent renders the flavanone a more potent antioxidant. Furthermore, chalcone **6** showed a moderate lipid peroxidation inhibition of 54.5%, similar to flavanone **4**.

### 3.3. Binding Studies with ct-DNA Using UV Spectroscopy

The interaction of the synthesized compounds with *calf thymus*-DNA was determined via UV spectroscopy. This method is based on the delocalization of the ctDNA band, located at 260–280 nm, in the presence of increasing amounts of ctDNA, when the latter interacts with a chemical entity. Slight changes in the absorbance of ctDNA have been correlated with the different types of binding modes [35]. More specifically, when DNA binds with compounds that act as intercalators, hypochromism is usually observed, while on the other hand, hyperchromism is detected in the case where the compounds interact with ctDNA by groove binding or electrostatic interaction. Furthermore, researchers have reported that compounds that result in bathocromism, or red shift, i.e., shifting of the maximum absorption to greater wavelengths, tend to stabilize the double helix of DNA, whereas compounds that result to blue shift have the exactly opposite effect [37,38]. In this work, based upon the changes in absorbance, the binding constant K_b_ was determined for each compound via the Benesi–Hildebrand equation, and the results are presented in Table 2. The binding of Rhodamine B (minor groove binder) and methyl green (major groove binder) was also studied [49,50].

**Figure 5:**
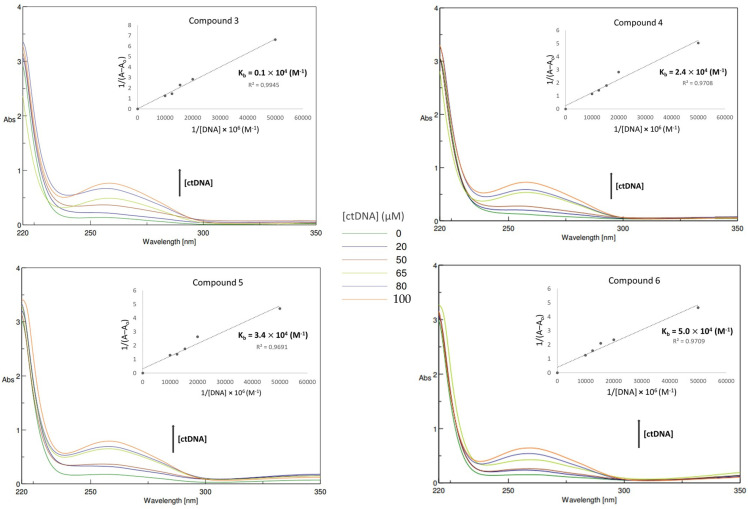
Absorption spectra of the synthesized compounds in tris−HCl with increasing concentration of ctDNA. Arrow refers to the hyperchromic effect.

The recorded spectra for the four synthesized compounds are depicted in Figure 5. Increasing amounts of ctDNA resulted in an increased absorption intensity (hyperchromism), followed by a red shift of the λ_max_, for all of the tested compounds, suggesting the formation of a ctDNA binding complex and the stabilization of ctDNA. According to the binding studies, binding constants range from 0.1 × 10^−3^ to 5.0 × 10^−3^ M^−1^, which indicates a moderate interaction of the compounds with the macromolecule. Among the tested compounds, chalcone **6** demonstrated the strongest interaction with ctDNA (K_b_ = 5 × 10^−3^ M^−1^), followed by arylidene flavanone **5** (K_b_ = 3.4 × 10^−3^ M^−1^), while the presence of a bromo substituent in the flavanone moiety (**3**) significantly reduces the binding constant (K_b_ = 0.1 × 10^−3^ M^−1^), and thus the interaction with ctDNA, in comparison with flavanone **4**, bearing a chloro-substituent (K_b_ = 0.24 × 10^−3^ M^−1^).

### 3.4. Molecular Docking

Molecular docking plays a crucial role in drug design that facilitates in the minimization of the cost and the experiments needed for the development of compounds with pharmaceutical properties and in a better understanding of bioactivity mechanisms. In an attempt to comprehend the mode of interaction and the binding affinity of the synthesized compounds, molecular docking studies were employed in which the DNA-compound complex is simulated, selecting the conformer with the minimum binding energy each time.

**Figure 6:**
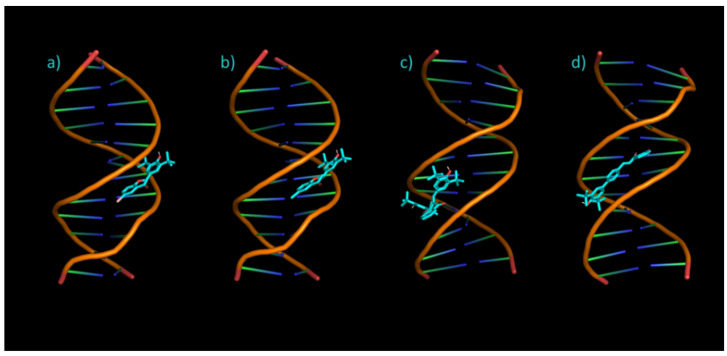
Docked pose of flavanones **3** (**a**) and **4** (**b**), arylidene flavanone **5** (**c**), and chalcone **6** (**d**) with B–DNA.

The docked complexes are shown in Figure 6. It is observed that all of the synthesized compounds bind in the major groove region of the DNA, and especially in the A-T-rich region. The simulated binding energy for the synthesized compounds is presented in Table 3. The negative sign depicts that the interaction of the compounds with DNA is spontaneous and thermodynamically favored.

**Figure 7:**
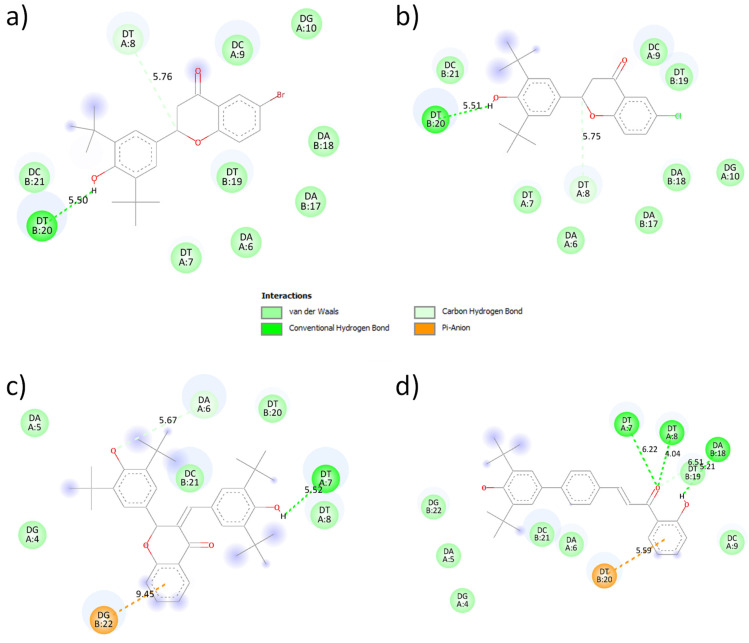
Docked interactions of flavanones **3** (**a**) and **4** (**b**), arylidene flavanone **5** (**c**), and chalcone **6** (**d**) with B-DNA.

The docked complex of flavanone **3**-DNA and its docked interactions are demonstrated in Figure 6a and Figure 7a, respectively. In the complex, the hydrogen of the hydroxyl group of the 3,5-di-*tert*-butyl-4-hydroxyphenyl substituent forms a hydrogen bond with the nucleotide B:DT20 at a distance of 5.50 Å, while carbon C-2 of the flavanone moiety participates in a carbon-hydrogen bond with nucleotide A:DT8 at a distance of 5.76 Å.

Similar results are observed for the docked complex of flavanone **4**-DNA and its docked interactions, presented in Figure 6b and Figure 7b, respectively. In the docked complex, the hydrogen of the hydroxyl group of the 3,5-di-*tert*-butyl-4-hydroxyphenyl substituent forms a hydrogen bond with nucleotide B:DT20 at a distance of 5.51 Å, while carbon C-2 of the flavanone moiety participates in a carbon-hydrogen bond with the nucleotide A:DT8 at a distance of 5.75 Å.

The docked complex of arylidene flavanone **5**-DNA and its docked interactions are demonstrated in Figure 6c and Figure 7c, respectively. In the docked complex, the oxygen of the hydroxyl group of the 3,5-di-*tert*-butyl-4-hydroxyphenyl substituent connected to C-2 and the hydrogen of the hydroxyl group of the BHT moiety connected to C-3 interact with a carbon-hydrogen bond and a hydrogen bond with nucleotides A:DA6 at a distance of 5.67 Å and A:DT7 at a distance of 5.52 Å, respectively. In addition to that, the aromatic ring A of the arylidene flavanone interacts electrostatically with nucleotide B:DG22 at a distance of 9.45 Å, a pi-anion bond, i.e., the non-covalent interaction between a π-acidic aromatic system and an anion.

The docked complex of chalcone **6**-DNA and its docked interactions are demonstrated in Figure 6d and Figure 7d, respectively. In the complex, the hydrogen of the 2′-hydroxyl group of the chalcone has a hydrogen bond interaction with the nucleotide B:DA18 at a distance of 5.21 Å. Furthermore, the carbonyl oxygen interacts with two hydrogen bonds with the nucleotides A:DT7 and A:DT8 at distances of 6.22 and 4.04 Å, while it binds with the nucleotide B:DT19 via a carbon-hydrogen bond at a distance of 6.51 Å. In addition to that, the aromatic ring A of the chalcone interacts electrostatically with nucleotide B:DT20 at a distance of 5.59 Å via a pi-anion bond.

### 3.5. Conformational Analysis of the Synthesized Compounds

In an effort to explore the conformational features of the novel flavanones (**3** and **4**), arylidene flavanone **5,** and chalcone **6,** theoretical calculations were applied, including random and systematic conformational searches. Specifically, the flavanones **3** and **4** (including their enantiomeric forms R and S), were subjected to energy minimization, followed by a random sampling conformational search. The conformational space of the flavanones **3** and **4** depends on the orientation of the 3,5 di-*tert*-butyl-4-hydroxyphenyl ring relative to the heterocyclic ring, as well as the rotation of the hydroxyl groups.

**Figure 8:**
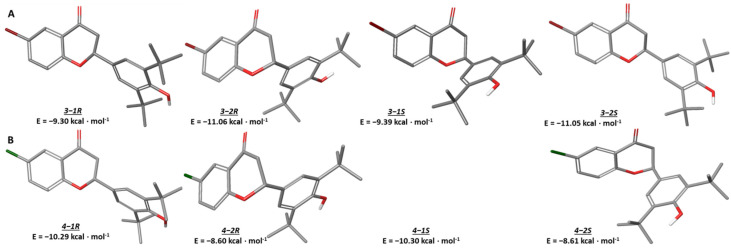
Representative low energy conformers of (**A**) compound **3** and (**B**) compound **4**.

**Figure A1:**
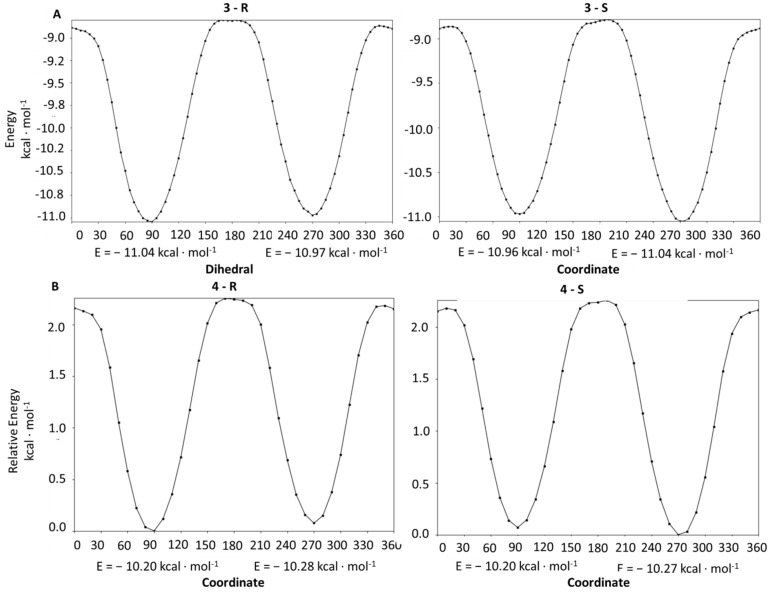
Energy minimum plots of (**A**) compound **3** (R,S) and (**B**) compound **4** (R,S) conformations, as obtained from the application of coordinate scan to the τ_1_ dihedral [41].

The generated conformers of each stereoisomer were classified into low energy clusters according to the number of their heavy atoms. In continuation, the members of each cluster that indicate the lowest energy values were further explored. In the case of compound **3**, two favorable conformations for both enantiomeric forms, namely **3**-1R, **3**-2R, **3**-1S, and **3**-2S, were generated and are illustrated in Figure 8A. The results indicated that all low energy conformers adopt an extended conformation without any formation of intramolecular hydrogen bonds. Similar results were obtained for compound **4,** where its lowest energy conformers are depicted in Figure 8B. In a step further, the torsional angle τ_1_ was studied for the flavanones **3** and **4** using systematic search (coordinate scan—macromodel) on the lowest energy conformers [42]. The results indicate two energetic minima (90° and 270°, Appendix A Figure A1), which confirm the extended conformation mentioned above.

**Figure 9:**
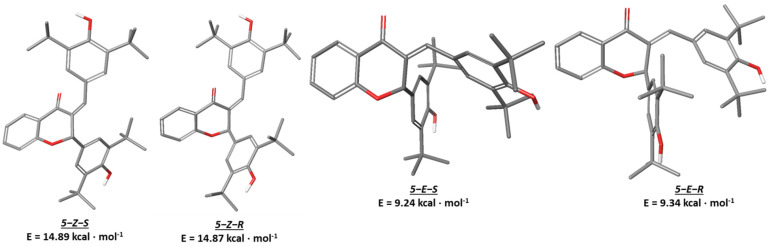
Representative low energy conformers of compound **5**.

In the case of arylidene flavanone **5**, the existence of a chiral center (2-position) and of a double bond, leading to four low energy conformations groups (**5**-Z-S, **5**-Z-R, **5**-E-S, and **5**-E-R), produced from a random sampling conformation search (Figure 9). The results indicated that in both *cis*-type low energy conformers (**5**-Z-S and **5**-Z-R), the aromatic ring D is oriented towards the carbonyl (-CO) group of flavanone ring C, while the aromatic ring B is in spatial proximity with the oxygen (-O) atom of the flavanone ring C. On the other hand, in both *trans*-type low energy conformers (**5**-E-S and **5**-E-Z), the aromatic rings D and B are placed in a spatial vicinity (Figure 9). Additionally, the comparison of the energy value between *trans* and *cis* type conformers highlights the crucial role of *trans*-type double bond.

**Figure 10:**
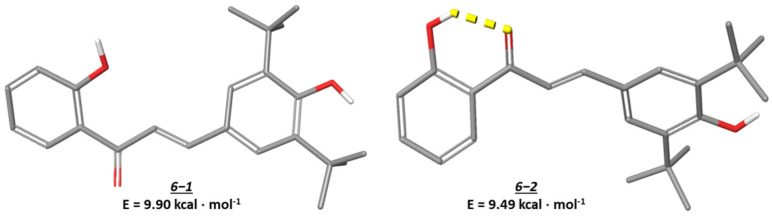
Representative low energy conformers of compound **6**. The intramolecular H-bond is depicted with yellow lines.

The described procedure was repeated for the synthesized chalcone **6** and the proposed low energy conformers are presented in Figure 10. Two favorable low energy conformations were proposed, both belonging to the S-*cis*-type of the chalcone conformers. Particularly, in the first conformer, the hydroxyl (-OH) group of ring A and the carbonyl (-CO) group are placed on the opposite side, while in the second conformer, a strong intramolecular H-bond is formed between the hydroxyl (-OH) group of ring A and the carbonyl group of the chalcone. This finding is in accordance with ^1^H NMR spectroscopy results for chalcone **6**, where the 2′-OH proton appears as a sharp singlet at 12.97 ppm, indicating a significant deshielding due to hydrogen bond formation. Results are also in accordance with a previous conformational analysis study on the bioactive flavanone naringenin [51].

**Figure 11:**
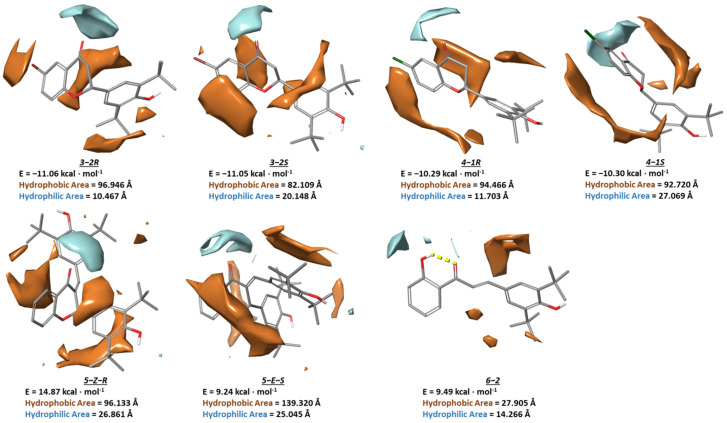
Hydrophobic (brown) and hydrophilic (blue) surface area representation for the lowest energy conformers of synthesized flavanones **3** and **4**, arylidene flavanone **5,** and chalcone **6** [41].

Finally, the generated polar surface maps presented higher hydrophobic (brown) areas compared to hydrophilic (blue) ones for the case of flavanones [44] and arylidene flavanone, which may improve the cellular permeability and biological response of these molecules, while the hydrophobic/philic maps of chalcone **6** exhibit a more amphoteric character (Figure 11).

### 3.6. Calculation of Physicochemical Properties

The physicochemical properties of the studied flavonoids analogues were predicted, utilizing the Qikprop program [35] of Schrödinger Suite (Release 2020-3) in order to unravel the crucial (and also conformation dependent) properties which can be used as descriptors for QSAR analysis. The calculated properties are presented in Table 4.

(a) Computed dipole moment of the molecule (dipole); (b) total solvent accessible surface area (SASA) in square angstroms using a probe with a 1.4 Å radius, (c) hydrophobic component of the SASA (FOSA); (d) hydrophilic component of the SASA (FISA); (e) p component of the SASA (PISA); (f) weakly polar component of the SASA (halogens, P, and S) (WPSA); (g) total solvent-accessible volume in cubic angstroms using a probe with a 1.4 Å radius (volume); (h) estimated average number of hydrogen bonds (taken over a number of configurations) that would be donated by the solute to water molecules in an aqueous solution (HBd); (i) estimated average number of hydrogen bonds (taken over a number of configurations) that would be accepted by the solute from water molecules in an aqueous solution (HBa); (j) square of the dipole moment divided by the molecular volume (dip^2^/V), a relevant parameter for the energy of solvation of a dipole of volume V; (k) hexadecane/gas partition coefficient (QPlogPC16); (l) octanol/gas partition coefficient (QPlogPoct); (m) water/gas partition coefficient (QPlogPw); (n) octanol/water partition coefficient (QPlogPo/w); (o) aqueous solubility in mol dm_3 (QPlogS); (p) conformation-independent predicted aqueous solubility (CIQPlogS); (q) PM3 calculated ionization potential (IP(ev)); (r) PM3 calculated electron affinity (EA(eV)); (s) van der Waals surface area of polar nitrogen and oxygen atoms (PSA).

## 4. Conclusions

In this work, two novel flavanones (**3** and **4**), arylidene flavanone **5,** and chalcone (**6**) were synthesized via an aldol reaction between 2-hydroxy-acetophenones (**1a**–**1c**) and 3,5-di-*tert*-butyl-4-hydroxybenzaldehyde (**2**) in acidic conditions. The synthesized compounds were evaluated for their antioxidant activity, as well as their interaction with ctDNA. The results of this study indicated that the presence of the 3,5–di–*tert*–butyl–4–hydroxyphenyl substituent contributes to the enhanced antioxidant activity of the flavanone framework. The ctDNA binding studies revealed that all the synthesized compounds tend to stabilize the double helix of the DNA, with chalcone **6** demonstrating the highest binding constant value, and thus the strongest interaction with ctDNA. A more in-depth study of the binding mechanism of the compounds to DNA indicated that the tested compounds bind in the major groove region of DNA and especially at the A-T-rich region, mainly via hydrogen bonds, van der Waals interactions, and electrostatic interactions. Furthermore, according to the analysis of conformational data, all low energy conformers of flavanones **3** and **4** adopted an extended conformation without forming any intramolecular bond. On the other hand, in the case of chalcone **6,** the conformational analysis indicated that the 2′-OH interacts with the -CO group of the α,β-unsaturated carbonyl moiety, forming an intramolecular hydrogen bond.

## Figures and Tables

**Table 1:** In vitro antioxidant evaluation of flavanones **3** and **4**, arylidene flavanone **5,** and chalcone **6**, through the DPPH scavenging ability and the inhibition of linoleic acid lipid peroxidation assays.

Compound	Structure	% Inhibition of DPPH Free Radical (100μM)		% Inhibition of Lipid Peroxidation of Linoleic Acid Induced by AAPH Radical (100 μM)
		30 min	60 min	
**3**	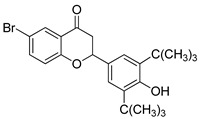	29.3	28.2	21.1
**4**	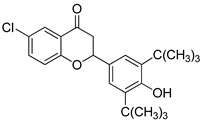	32.0	39.6	59.3
**5**	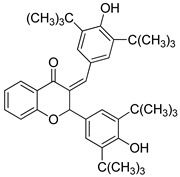	70.8	60.2	77.4
**6**	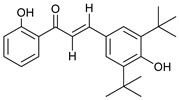	61.1	39.9	54.6
TROLOX		82.4	83.4	81.4

**Table 2:** Binding constants of the synthesized compounds.

Compound	Structure	Κ_b_ (M^−1^)
**3**	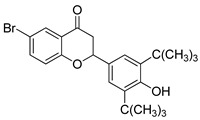	0.1 × 10^3^
**4**	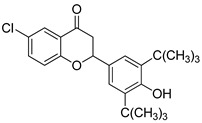	2.4 × 10^3^
**5**	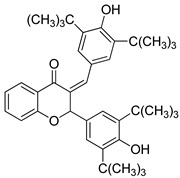	3.4 × 10^3^
**6**	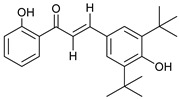	5.0 × 10^3^
Rhodamine B		2.2 × 10^4^
Methyl Green		2.0 × 10^3^

**Table 3:** Docking energy values for the synthesized compounds-DNA complexes.

Compound	Binding Energy (kcal/mol)	Inhibition Constant K_i_	vdW + Bond + Desolve Energy (kcal/mol)	Electrostatic Energy (kcal/mol)
**3**	−9.01	250.10 nM	−10.10	−0.10
**4**	−8.97	316.49 nM	−9.97	−0.09
**5**	−6.35	22.11 μM	−8.64	−0.10
**6**	−9.06	229.64 nM	−11.52	+0.07

**Table 4:** Physicochemical properties of the flavanones **3** and **4**, the arylidene flavanone **5,** and the chalcone **6**.

Physicochemical Property	Compounds			
	**3**	**4**	**5**	**6**
Dipole	4.97	5.26	5.87	4.81
SASA	687.00	681.97	893.34	680.30
FOSA	412.17	412.12	642.62	378.37
FISA	57.75	57.76	49.28	90.75
PISA	139.80	140.52	201.43	211.18
WPSA	77.33	71.58	0	0
volume	1256.42	1247.51	1852.64	1231.98
donorHB	1	1	2	1
accptHB	3.5	3.5	4.25	2.5
dip^2^/V	0.019641	0.0222095	0.0186334	0.0187806
QPpolrz	43.54	43.19	64.77	40.03
QPlogPC16	12.06	11.94	17.30	12.02
QPlogPoct	17.94	17.83	26.99	15.87
QPlogPw	6.99	6.98	8.92	5.57
QPlogPo/w	5.77	5.69	8.90	5.60
QPlogS	−7.36	−7.24	−9.49	−6.46
IP(eV)	9.04	9.03	8.88	8.86
EA(eV)	0.79	0.77	0.71	0.88
PSA	47.40	47.40	57.11	58.20

## Data Availability

The Data presented in the study are available on request from the corresponding author.

## Supplementary Materials

The supplementary materials are available online at https://www.mdpi.com/article/10.3390/antiox11112273/s1.

## References

[1] Zhuang C., Zhang W., Sheng C., Zhang W., Xing C., Miao Z. (2017). Chalcone: A Privileged Structure in Medicinal Chemistry. Chem. Rev..

[2] Kostopoulou I., Detsi A. (2018). Recent Developments on Tyrosinase Inhibitors based on the Chalcone and Aurone Scaffolds. Curr. Enzym. Inhib..

[3] Detsi A., Majdalani M., Kontogiorgis C.A., Hadjipavlou-Litina D., Kefalas P. (2009). Natural and synthetic 2′-hydroxy-chalcones and aurones: Synthesis, characterization and evaluation of the antioxidant and soybean lipoxygenase inhibitory activity. Bioorg. Med. Chem..

[4] Kostopoulou I., Tzani A., Polyzos N.-I., Karadendrou M.-A., Kritsi E., Pontiki E., Liargkova T., Hadjipavlou-Litina D., Zoumpoulakis P., Detsi A. (2021). Exploring the 2-Hydroxy-Chalcone Framework for the Development of Dual Antioxidant and Soybean Lipoxygenase Inhibitory Agents. Molecules.

[5] Santi M.D., Peralta M.A., Mendoza C.S., Cabrera J.L., Ortega M.G. (2017). Chemical and bioactivity of flavanones obtained from roots of *Dalea pazensis* Rusby. Bioorg. Med. Chem. Lett..

[6] Cheenpracha S., Pyne S.G., Patrick B.O., Andersen R.J., Maneerat W., Laphookhieo S. (2019). Mallopenins A-E, Antibacterial Phenolic Derivatives from the Fruits of *Mallotus philippensis*. J. Nat. Prod..

[7] Khan M.K., Zill-E-Huma, Dangles O. (2014). A comprehensive review on flavanones, the major citrus polyphenols. J. Food Compos. Anal..

[8] Wang S.W., Sheng H., Bai Y.F., Weng Y.Y., Fan X.Y., Zheng F., Fu J.Q., Zhang F. (2021). Inhibition of histone acetyltransferase by naringenin and hesperetin suppresses Txnip expression and protects pancreatic β cells in diabetic mice: Naringenin and hesperetin protect pancreatic β cells. Phytomedicine.

[9] Yang J., Liu L., Li M., Huang X., Yang H., Li K. (2021). Naringenin inhibits pro-inflammatory cytokine production in macrophages through inducing MT1G to suppress the activation of NF-κB. Mol. Immunol..

[10] Pontifex M.G., Malik M.M.A.H., Connell E., Müller M., Vauzour D. (2021). Citrus Polyphenols in Brain Health and Disease: Current Perspectives. Front. Neurosci..

[11] Justino G.C., Rodrigues M., Florêncio M.H., Mira L. (2009). Structure and antioxidant activity of brominated flavonols and flavanones. J. Mass Spectrom..

[12] Özyürek M., Akpınar D., Bener M., Türkkan B., Güçlü K., Apak R. (2014). Novel oxime based flavanone, naringin-oxime: Synthesis, characterization and screening for antioxidant activity. Chem. Biol. Interact..

[13] Di Majo D., Giammanco M., La Guardia M., Tripoli E., Giammanco S., Finotti E. (2005). Flavanones in Citrus fruit: Structure–antioxidant activity relationships. Int. Food Res. J..

[14] Silva M.M., Santos M.R., Caroço G., Rocha R., Justino G., Mira L. (2002). Structure-antioxidant activity relationships of flavonoids: A re-examination. Free Radic. Res..

[15] Tajammala A., Batoola M., Ramzana A., Samraa M.M., Mahnoora I., Verpoortb F., Irfancd A., Al-Sehemicd A.G., Munawara M.A., Basra M.A.R. (2017). Synthesis, antihyperglycemic activity and computational studies of antioxidant chalcones and flavanones derived from 2, 5 dihydroxyacetophenone. J. Mol. Struct..

[16] Lee J.H. (2015). In-vitro evaluation for antioxidant and anti-inflammatory property of flavanone derivatives. Food Biosci..

[17] Rice-Evans C.A., Miller N.J., Paganga G. (1996). Structure-antioxidant activity relationships of flavonoids and phenolic acids. Free Radic. Biol. Med..

[18] Ahmed S., Shakeel F. (2012). Antioxidant activity coefficient, mechanism, and kinetics of different derivatives of flavones and flavanones towards superoxide radical. Czech J. Food Sci..

[19] Chuang S.Y., Lin Y.K., Lin C.F., Wang P.W., Chen E.L., Fang J.Y. (2017). Elucidating the skin delivery of aglycone and glycoside flavonoids: How the structures affect cutaneous absorption. Nutrients.

[20] Speisky H., Shahidi F., Costa de Camargo A., Fuentes J. (2022). Revisiting the oxidation of flavonoids: Loss, conservation or enhancement of their antioxidant properties. Antioxidants.

[21] Mira L., Tereza Fernandez M., Santos M., Rocha R., Helena Florêncio M., Jennings K.R. (2002). Interactions of flavonoids with iron and copper ions: A mechanism for their antioxidant activity. Free Radic. Res..

[22] Hrelia S., Angeloni C. (2020). New mechanisms of action of natural antioxidants in health and disease. Antioxidants.

[23] Procházková D., Boušová I., Wilhelmová N. (2011). Antioxidant and prooxidant properties of flavonoids. Fitoterapia.

[24] De Souza Farias S.A., da Costa K.S., Martins J.B. (2021). Analysis of conformational, structural, magnetic, and electronic properties related to antioxidant activity: Revisiting flavan, anthocyanidin, flavanone, flavonol, isoflavone, flavone, and flavan-3-ol. ACS Omega.

[25] Mottaghipisheh J., Stuppner H. (2021). A comprehensive review on chemotaxonomic and phytochemical aspects of homoisoflavonoids, as rare flavonoid derivatives. Int. J. Mol. Sci..

[26] Jeong H.J., Kim Y.M., Kim J.H., Kim J.Y., Park J.Y., Park S.J., Ryu Y.B., Lee W.S. (2012). Homoisoflavonoids from *Caesalpinia sappan* displaying viral neuraminidases inhibition. Biol. Pharm. Bull..

[27] Cuong T.D., Hung T.M., Kim J.C., Kim E.H., Woo M.H., Choi J.S., Lee J.H., Min B.S. (2012). Phenolic compounds from *caesalpinia sappan* heartwood and their anti-inflammatory activity. J. Nat. Prod..

[28] Siddaiah V., Rao C.V., Venkateswarlu S., Krishnaraju A.V., Subbaraju G.V. (2006). Synthesis, stereochemical assignments, and biological activities of homoisoflavonoids. Bioorganic Med. Chem..

[29] Zhao J., Zhu A., Sun Y., Zhang W., Zhang T., Gao Y., Shan D., Wang S., Li G., Zeng K. (2020). Beneficial effects of sappanone A on lifespan and thermotolerance in Caenorhabditis elegans. Eur. J. Pharmacol..

[30] Roy S.K., Kumari N., Gupta S., Pahwa S., Nandanwar H., Jachak S.M. (2013). 7-Hydroxy-(E)-3-phenylmethylene-chroman-4-one analogues as efflux pump inhibitors against *Mycobacterium smegmatis* mc2 155. Eur. J. Med. Chem..

[31] Roy S.K., Agrahari U.C., Gautam R., Srivastava A., Jachak S.M. (2012). Isointricatinol, a new antioxidant homoisoflavonoid from the roots of *Caesalpinia digyna* Rottler. Nat. Prod. Res..

[32] Yehye W.A., Rahman N.A., Ariffin A., Abd Hamid S.B., Alhadi A.A., Kadir F.A., Yaeghoobi M. (2015). Understanding the chemistry behind the antioxidant activities of butylated hydroxytoluene (BHT): A review. Eur. J. Med. Chem..

[33] Kostopoulou I., Diassakou A., Kavetsou E., Kritsi E., Zoumpoulakis P., Pontiki E., Hadjipavlou-Litina D., Detsi A. (2020). Novel quinolinone–pyrazoline hybrids: Synthesis and evaluation of antioxidant and lipoxygenase inhibitory activity. Mol. Divers..

[34] Hadjipavlou-Litina D., Garnelis T., Athanassopoulos C.M., Papaioannou D. (2009). Kukoamine A analogs with lipoxygenase inhibitory activity. J. Enzyme Inhib. Med. Chem..

[35] Sirajuddin M., Ali S., Badshah A. (2013). Drug-DNA interactions and their study by UV-Visible, fluorescence spectroscopies and cyclic voltametry. J. Photochem. Photobiol. B Biol..

[36] Shahabadi N., Fili S.M., Kheirdoosh F. (2013). Study on the interaction of the drug mesalamine with calf thymus DNA using molecular docking and spectroscopic techniques. J. Photochem. Photobiol. B Biol..

[37] Tzani A., Kalafateli S., Tatsis G., Bairaktari M., Kostopoulou I., Pontillo A.R., Detsi A. (2021). Natural Deep Eutectic Solvents (NaDESs) as Alternative Green Extraction Media for Ginger (*Zingiber officinale* Roscoe). Sustain. Chem..

[38] Pontillo A.R.N., Konstanteli E., Bairaktari M.M., Detsi A. (2021). Encapsulation of the natural product tyrosol in carbohydrate nanosystems and study of their binding with ctdna. Polymers.

[39] Adams J.H. (1967). Influence of bulky substituents on the syntheses of 4-hydroxy-3, 5-dialkyl flavonoids. J. Org. Chem..

[40] Katopodi A., Tsotsou E., Iliou T., Deligiannidou G.E., Pontiki E., Kontogiorgis C., Tsopelas F., Detsi A. (2021). Synthesis, bioactivity, pharmacokinetic and biomimetic properties of multi-substituted coumarin derivatives. Molecules.

[41] (2020). Schrödinger Release 2020-3: MacroModel.

[42] Jorgensen W.L., Maxwell D.S., Tirado-Rives J. (1996). Development and testing of the OPLS all-atom force field on conformational energetics and properties of organic liquids. J. Am. Chem. Soc..

[43] Polak E., Ribiere G. (1969). Revenue Francaise Informat. Rech. Oper..

[44] (2020). Schrödinger Release 2020-3: Maestro.

[45] (2020). Schrödinger Release 2020-3: QikProp.

[46] Enes R.F., Farinha A.S.F., Tomé A.C., Cavaleiro J.A.S., Amorati R., Petrucci S., Pedulli G.F. (2009). Synthesis and antioxidant activity of [60] fullerene-flavonoid conjugates. Tetrahedron.

[47] Zhang Y.L., Wang Y.Q. (2014). Enantioselective biomimetic cyclization of 2′-hydroxychalcones to flavanones. Tetrahedron Lett..

[48] Desai J., Nair K.B., Misra A.N. (2001). Synthesis and antimicrobial activities of some new pyrazolines phenyl pyrazolines, flavanones and related compounds. Indian J. Heterocycl. Chem..

[49] Islam M.M., Chakraborty M., Pandya P., Al Masum A., Gupta N., Mukhopadhyay S. (2013). Binding of DNA with Rhodamine B: Spectroscopic and molecular modeling studies. Dye. Pigment..

[50] Prieto D., Aparicio G., Morande P.E., Zolessi F.R. (2014). A fast, low cost, and highly efficient fluorescent DNA labeling method using methyl green. Histochem. Cell Biol..

[51] Ávila E.P., Mendes L.A.O., De Almeida W.B., Santos H.F.D., De Almeida M.V. (2021). Conformational analysis and reactivity of naringenin. J. Mol. Struct..

