# Supplementary material for: Synthesis, Conformational Analysis and ctDNA Binding Studies of Flavonoid Analogues Possessing the 3,5-di-tert-butyl-4-hydroxyphenyl Moiety
Source: Antioxidants (Basel). 2022 Nov 17;11(11):2273. doi: 10.3390/antiox11112273 (PMC9687229; doi:10.3390/antiox11112273)

^{1}H NMR of compound 3

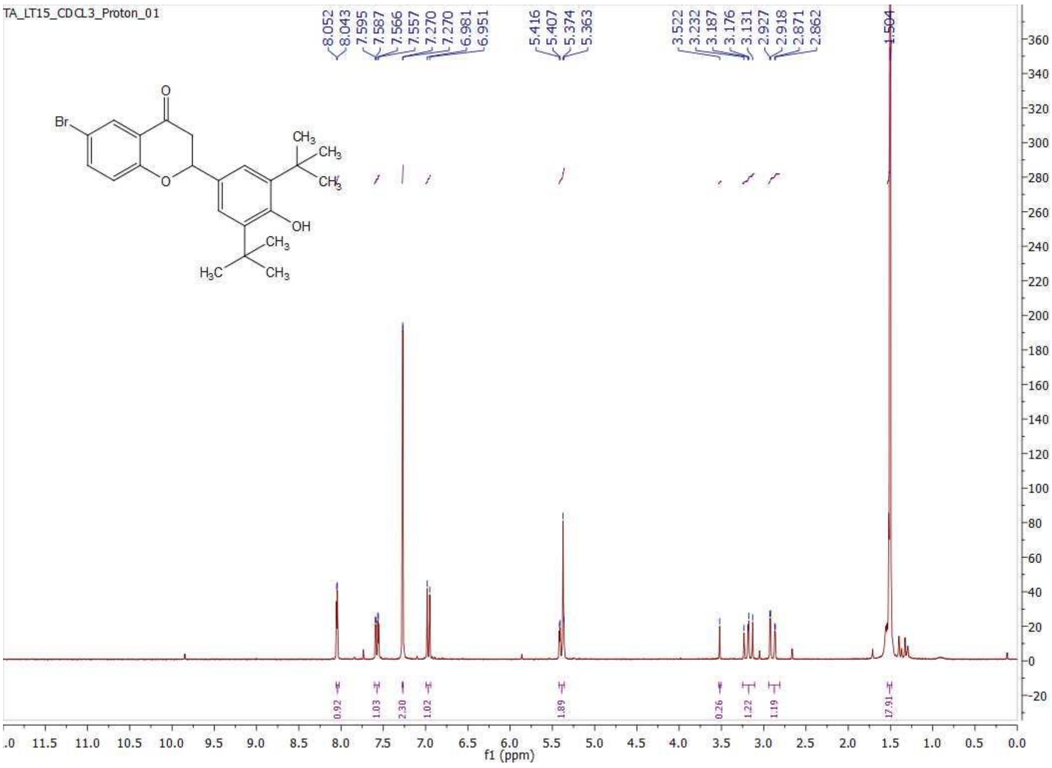

^{13}C NMR of compound 3

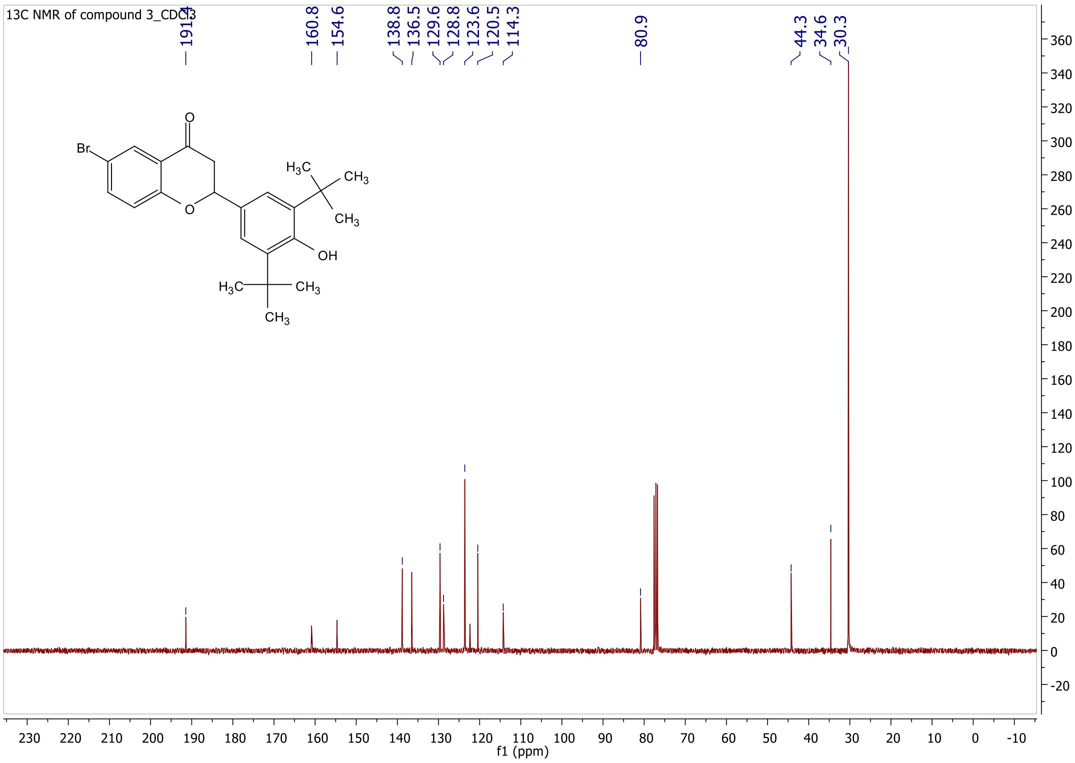

¹H NMR of compound 4

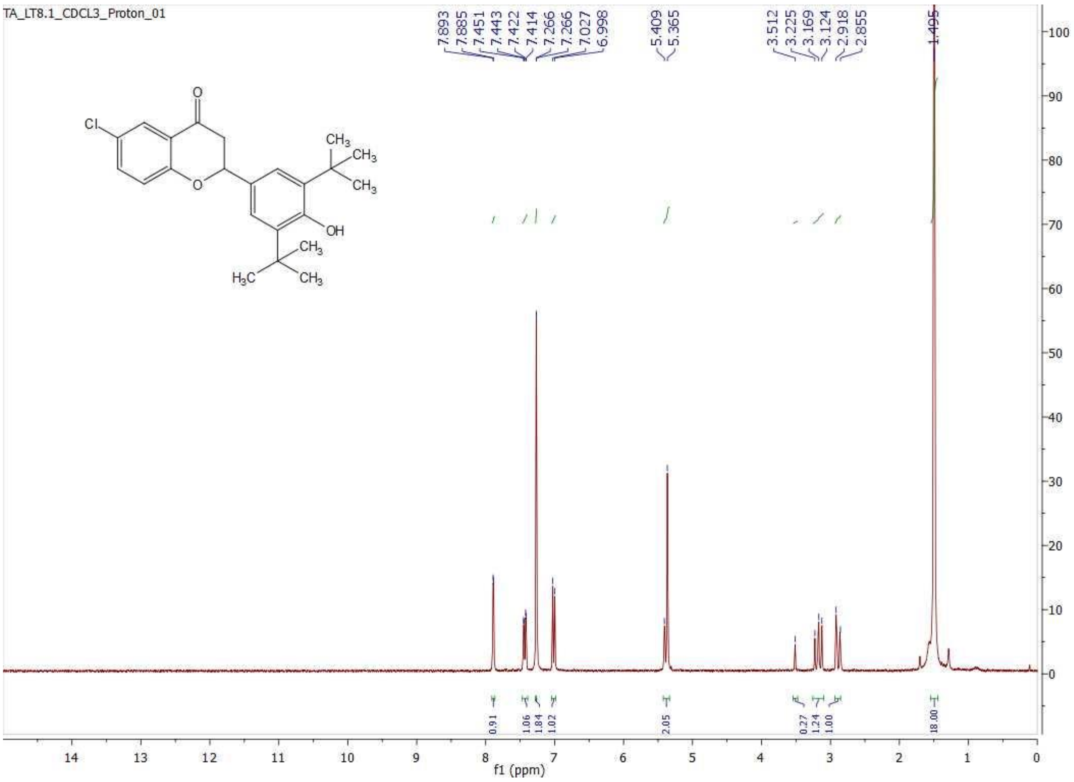

¹³C NMR of compound 4

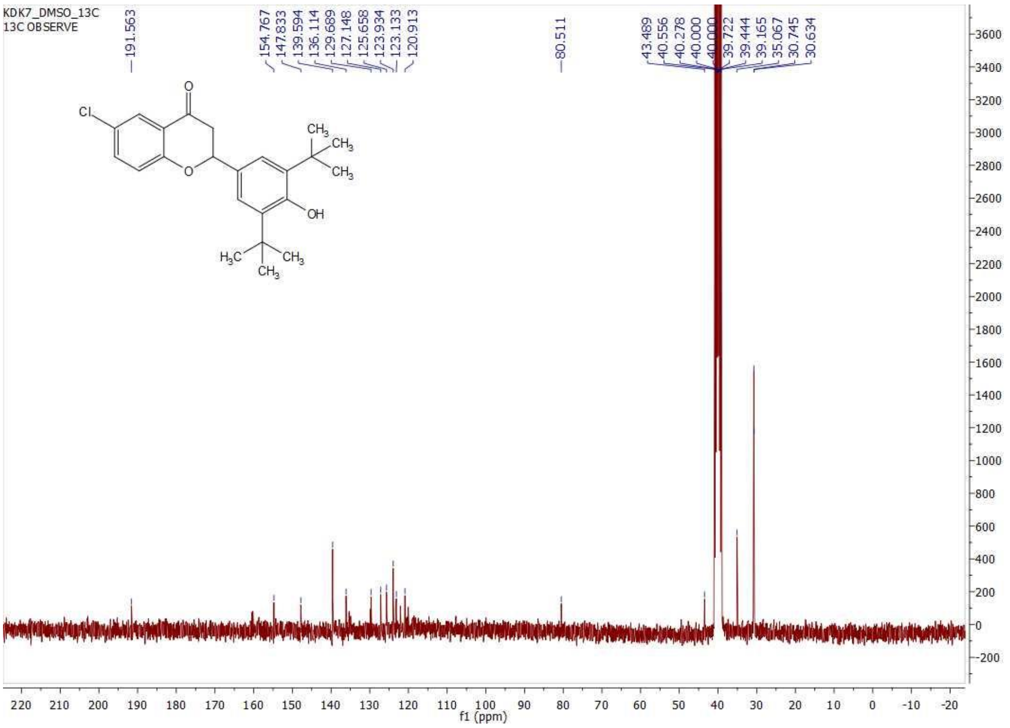

^{1}H NMR of compound 5

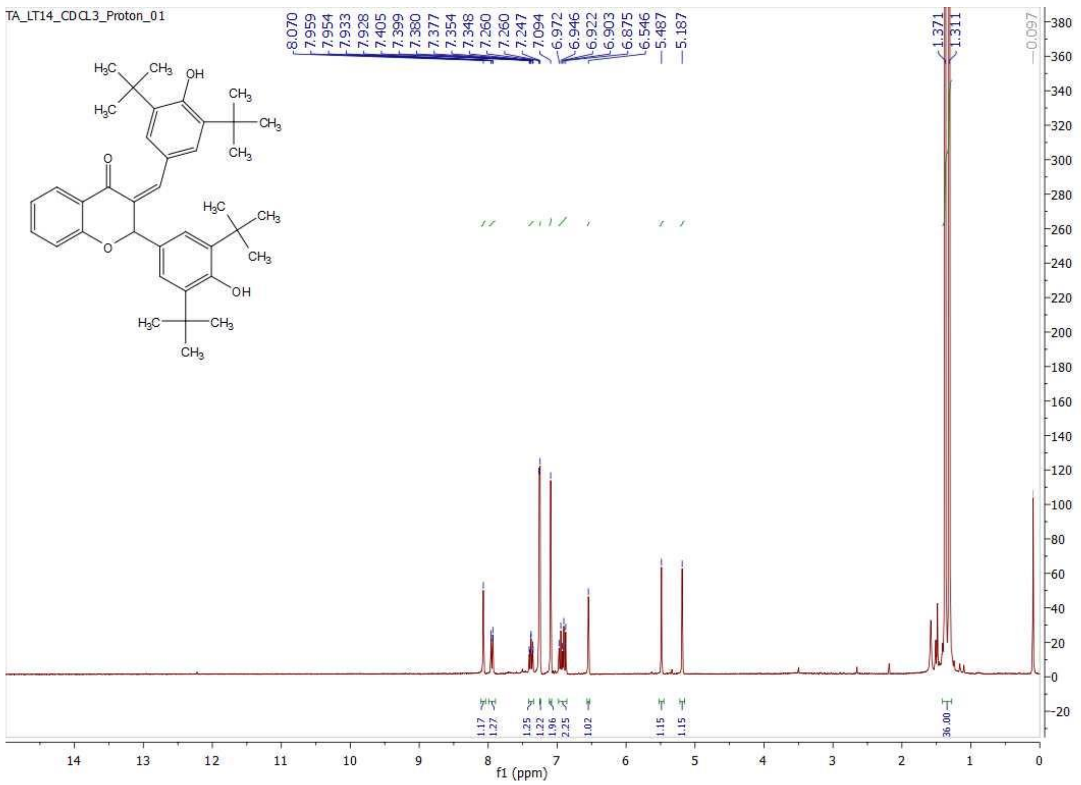

^{13}C NMR of compound 5

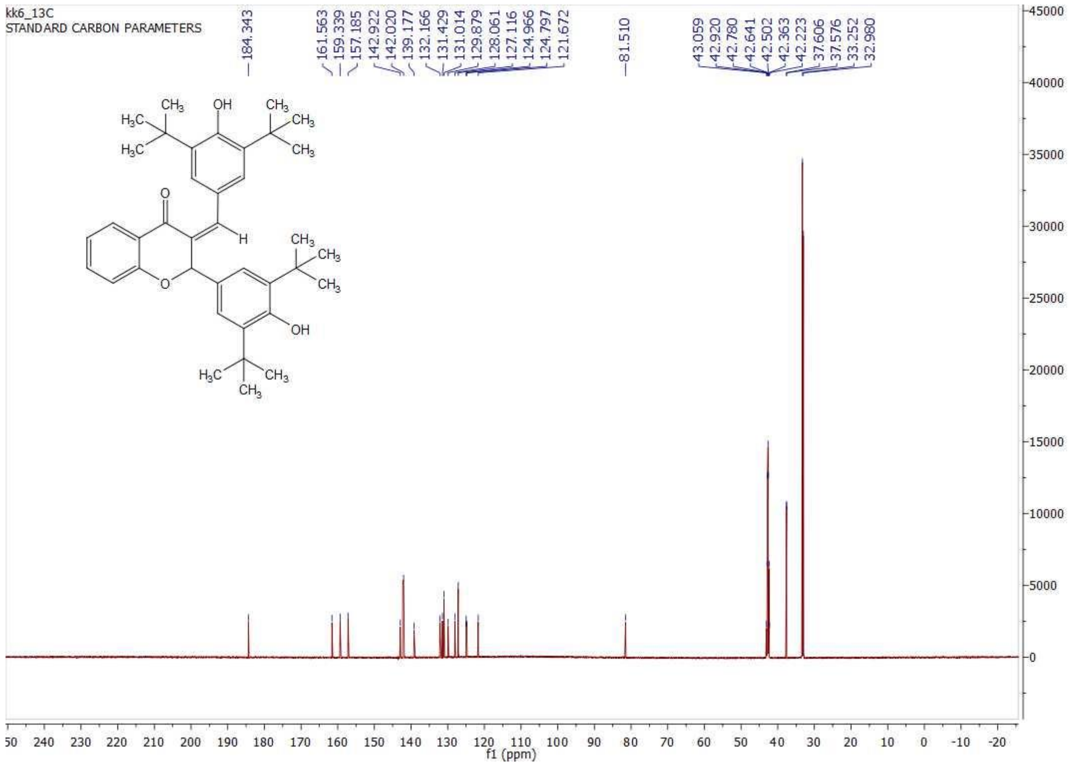

^{1}H NMR of compound 6

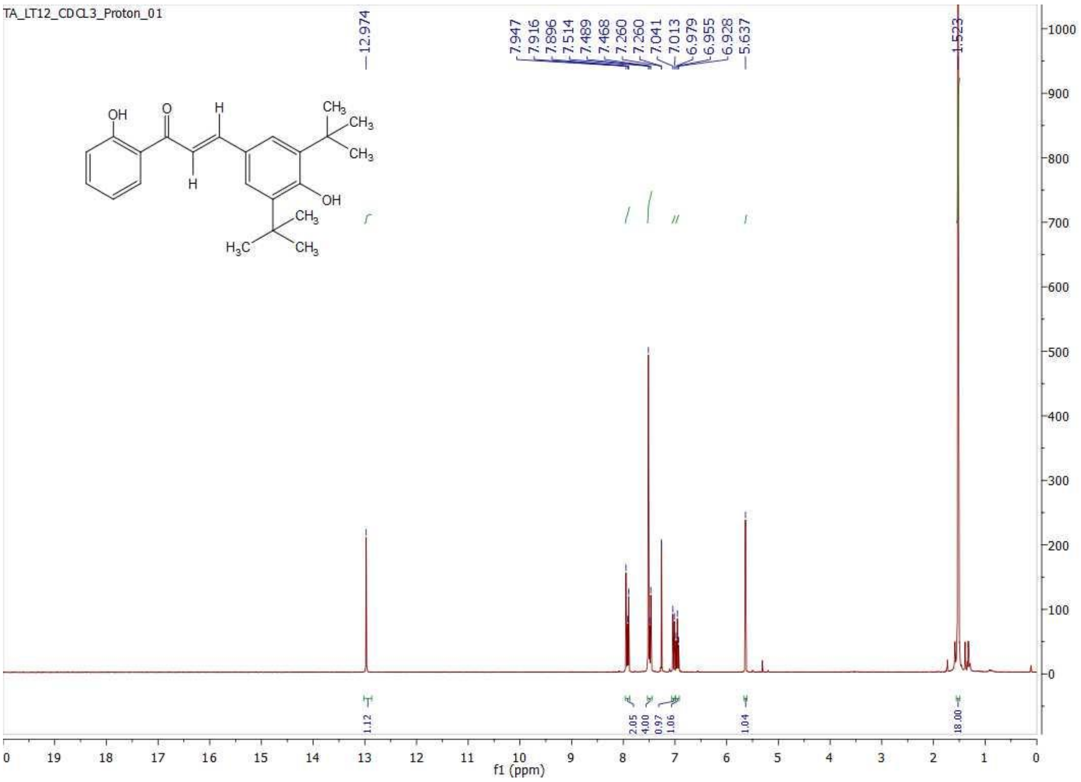

^{13}C NMR of compound 6

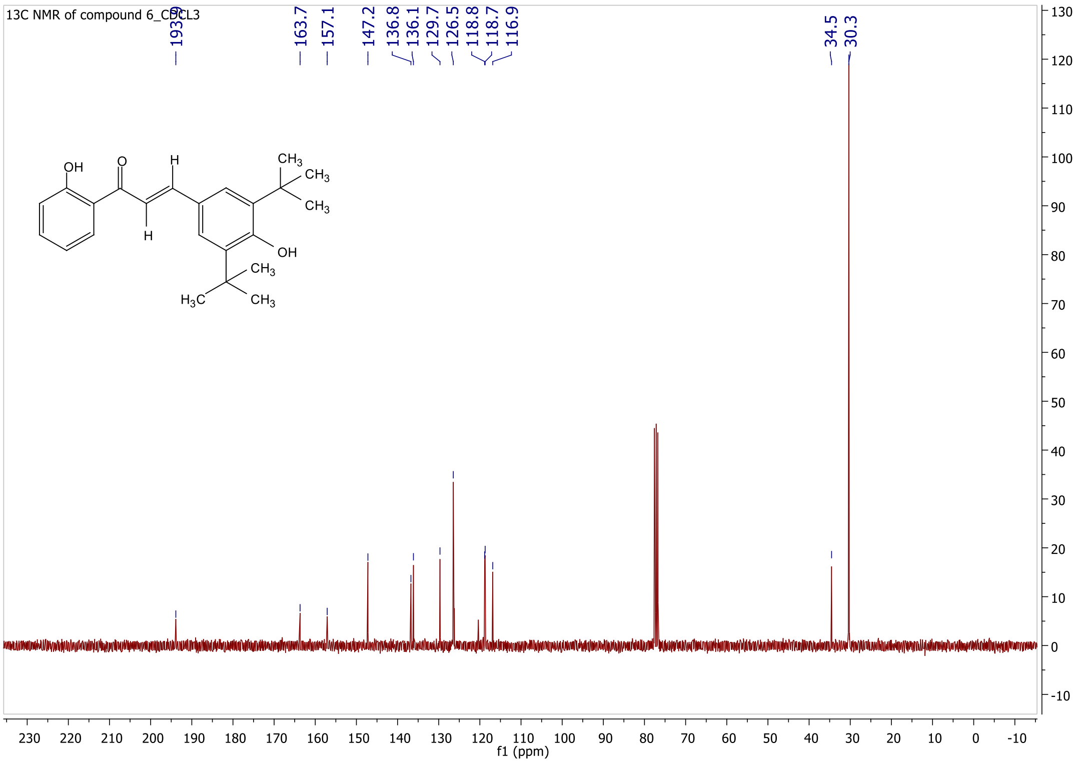

MS of compound 3

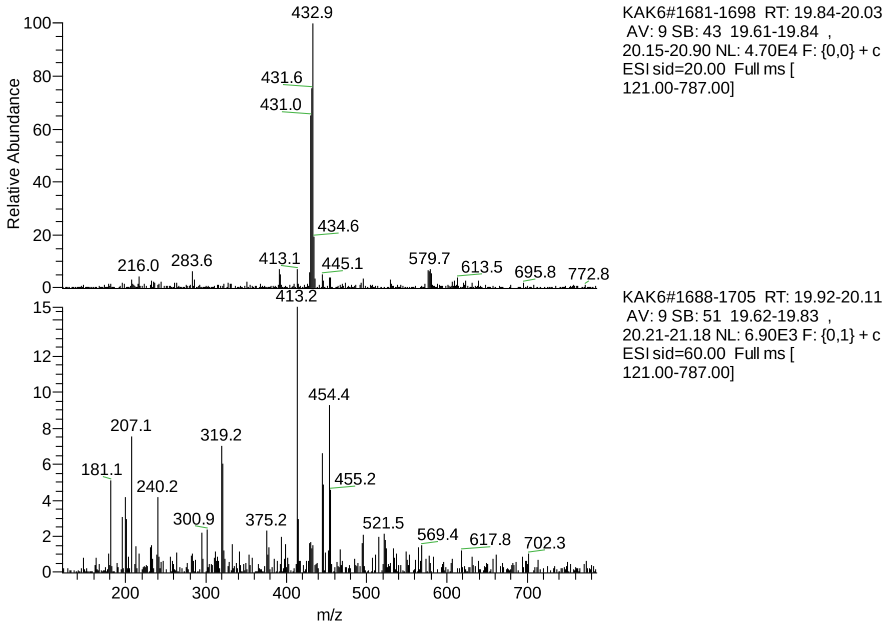

MS of compound 4

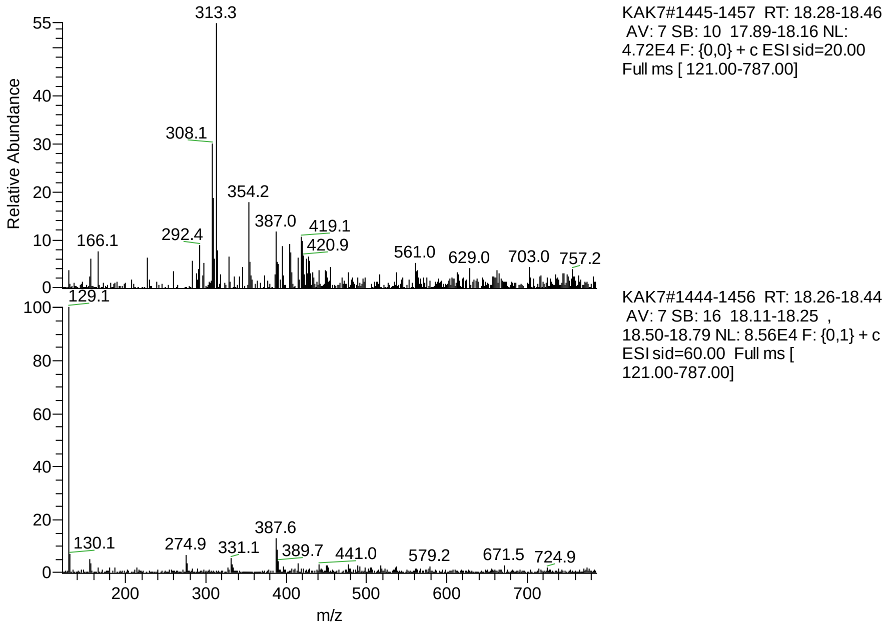

MS of compound 5

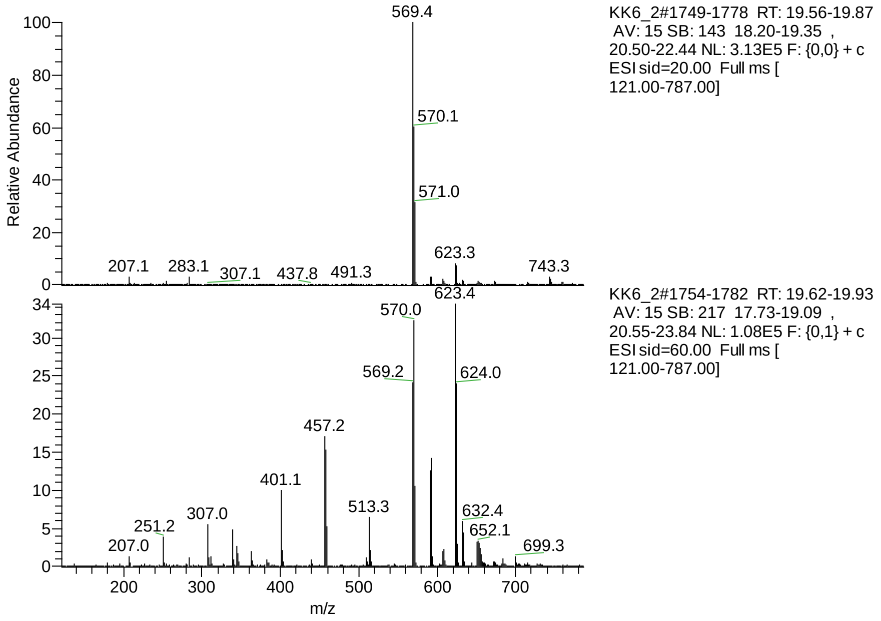

MS of compound 6

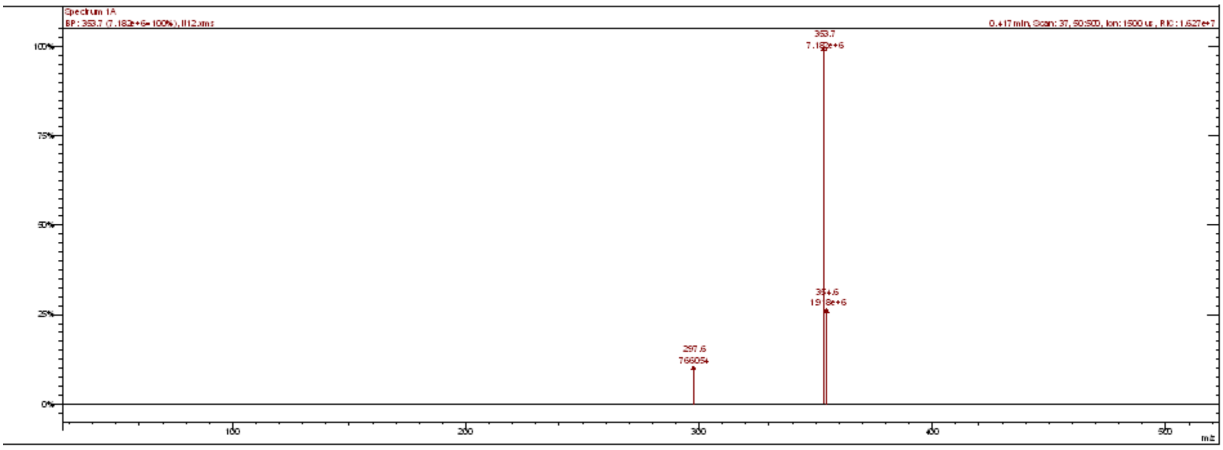

Supplement: Supplementary file 1 [file antioxidants-11-02273-s001.zip › antioxidants-2012732-supplementary.pdf]
